# Scorpion and centipede alleviates severe asthma through M2 macrophage-derived exosomal miR-30b-5p

**DOI:** 10.18632/aging.204053

**Published:** 2022-05-02

**Authors:** Binqing Tang, Yingen Wu, Yada Zhang, Yanqi Cheng, Yuqin Wu, Hong Fang

**Affiliations:** 1 Department of Respiratory Medicine, Shanghai Municipal Hospital of Traditional Chinese Medicine, Shanghai University of Traditional Chinese Medicine, Shanghai, China; 2 Prevention and Health Care Department of TCM, Longhua Hospital, Shanghai University of Traditional Chinese Medicine, Shanghai, China

**Keywords:** scorpion, centipede, M2 macrophage, exosome, miR-30b-5p, pyroptosis, asthma

## Abstract

Asthma is one of the most common chronic inflammatory diseases. Although the scorpion and centipede (SC) significantly ameliorates asthma and changes exosomal miRNAs, the molecular mechanism is still obscure. Here, we show that SC improves inflammation in asthmatic mice and increases M2 macrophage-derived exosomes (M2Φ-Exos) by promoting M2 macrophage polarization. The M2Φ-Exos remarkably inhibits airway epithelial cell pyroptosis by reducing the expression of NLRP3, caspase-1, and LI-1β and mitochondrial swelling. Furthermore, miR-30b-5p is up-regulated in M2Φ-Exos compared with M1Φ-Exos. Overexpression of miR-30b-5p in M2Φ-Exos prevents airway epithelial cell pyroptosis, while down-regulation of miR-30b-5p promotes pyroptosis. We also uncover that pyroptosis is increased in asthmatic mice, while SC blocks pyroptosis. Moreover, miR-30b-5p overexpressed M2Φ-Exos further enhances the ameliorative effect of SC, which significantly down-regulates IRF7 expression. Our results collectively reveal that M2Φ-Exos induced by SC could carry miR-30b-5p to mitigate severe asthma by inhibiting airway epithelial cell pyroptosis. Most importantly, our findings may provide a potential clinical application of M2Φ-Exos for treating severe asthma.

## INTRODUCTION

Asthma is a severe global health problem and is one of the most common chronic inflammatory diseases characterized by respiratory symptoms and restricted airflow [1]. In recent years, asthma prevalence and fatality rates have risen all over the world. Currently, there are around 300 million asthmatic patients globally, and the incidence of asthma in adults is 1%~2% [2]. Factors, such as allergens or irritants, respiratory tract infections, climate change, and stress, can contribute to differences in asthma symptoms and severity [3]. A small number of patients with asthma show severe disease progression and develop severe asthma (SA), which is poorly controlled and sometimes even endangers life. Although SA patients make up a small proportion of the total number of people with asthma (5%–10%), they account for 50% of total healthcare costs, making SA considerable health and socio-economic burden [4]. Insect Chinese medicine, such as scorpion and centipede (SC), is usually applied to treating SA [5]. In fact, scorpion toxins were also found to have a non-specific desensitization effect on patients with allergic rhinitis and asthma. It is well-known that SC can improve the level of clinical control, lung function and FeNO in patients with refractory asthma and significantly alleviated the pathogenesis of asthma by suppressing the release of inflammatory cytokines. We previously determined that SC reduced the inflammatory response in asthmatic mice [6]. Nevertheless, the specific mechanisms of SC therapy remain obscure. Further studies on the mechanisms of SC in the treatment of SA could contribute to developing new therapeutic methods for SA.

Exosomes are tiny vesicles with sizes between 30 and 100 nm in diameter and can be released by numerous cells, including macrophages, neutrophils, dendritic cells, and lymphocytes [7]. Exosomes mediate cell-to-cell communication by carrying various molecules, such as nucleic acids (DNA, mRNA, and miRNA), lipid, and proteins. Recent studies have shown that exosomes are involved in immune regulation in various diseases, including asthma, acute lung injury, and cancer [8–10]. Due to the unique molecular properties, exosomes can be loaded with different molecules and used as drug delivery vehicles, serving as novel therapeutic tools for asthma. A recent study reported that M2 macrophage-derived exosomes (M2Φ-Exos) carried miR-370 to mitigate asthma progression via restraining the FGF1/MAPK/STAT1 signaling axis [11]. Additionally, exosomes have been used as biomarkers for multiple diseases. Exosomal miR-125b may be used as a non-invasive diagnostic indicator for asthma severity with high diagnostic efficacy [12]. Our previous study found that SC could significantly change miRNA expression in exosomes of bronchoalveolar lavage fluid, thus improving the inflammatory symptoms of mice with asthma [6]. However, what kinds of exosomes are mainly mediated by SC, and the functions or molecular mechanisms of these exosomes involved in asthma are still unclear.

Pyroptosis is a newly discovered way of programmed inflammatory cell death [13]. Previous studies have demonstrated that pyroptosis played a central role in various diseases, including cancers, inflammatory diseases, nervous system diseases, and cardiovascular diseases [14–16]. The nod-like receptor protein-3 (NLRP3) inflammasome, a key molecule in the process of pyroptosis, may be a novel diagnostic or therapeutic target for inflammatory diseases [17]. Although NLRP3 inflammasomes are critical for providing protective immunity, over-activation of inflammasome-mediated response can cause excessive inflammation and tissue injury, thus leading to the development of chronic inflammatory diseases, especially SA [18]. A study suggested that bronchial epithelial pyroptosis aggravated airway inflammation and hyper-responsiveness in toluene diisocyanate-induced asthma [19]. Besides, exosome therapy could enhance anti-inflammatory M2 macrophages and reduce pyroptosis in doxorubicin-induced cardiomyopathy [20]. Our previous study suggested that the secretion of IL-1β, a marker of pyroptosis, was decreased during the treatment of SA by SC. However, it is unclear whether SC-induced exosomal miRNAs mediates pyroptosis in SA. The aim of this study is to identify which cells are the primary sources of SC-induced exosomes and explore how the SC-induced exosomes alleviate SA progression by regulating airway epithelial cell pyroptosis.

## MATERIALS AND METHODS

### Ethics statement

All experimental procedures were approved by the Ethical Committee and the Animal Experimental Committee of Shanghai Municipal Hospital of Traditional Chinese Medicine, Shanghai University of Traditional Chinese Medicine. The animal experiments involved in this study were performed according to the Guide for the Care and Use of Laboratory Animals of the National Institutes of Health.

### Establishment of asthma model and SC treatment

A total of 60 female BALB/c mice (weighing 18–22 g) aging 8-12-week old were purchased from Shanghai Laboratory Animal Center (Shanghai, China). All mice were housed in plastic cages and given free access to food and water. The mice were randomly allocated to three groups: sham, SA, and SC. The establishment of the asthma model was performed as reported previously [6]. During the asthma sensitization, mice in the SA and SC groups were intraperitoneally injected with 0.1 ml of saline solution containing OVA (0.5 mg/mL, Sigma, cat#A5503) and aluminum hydroxide (2 mg/mL, Sigma, V900163). From the 15^th^ day, mice were placed in a closed container filled with 2% OVA atomized solution for 40 min every day. The mice in the control group were intraperitoneally injected with saline solution on the 1^st^ and 7^th^ days and placed in a closed container filled with the saline atomized solution on the 15^th^ day. On 15–36 days, the control group and asthma group received 10 mL/kg saline by intragastric administration before 1 h of each stimulation, and the SC treatment group received SC solution at a dose of 0.625 g/kg. Finally, mice were euthanized via CO_2_ inhalation and collected bronchoalveolar lavage fluid (BALF) and lung tissues.

### Isolation of macrophages and polarization of M1 and M2 macrophages

To identify the effect of SC on macrophages, primary macrophages were isolated from the BALF of mice [21]. The isolation of macrophages was performed according to the methods in previous studies. In brief, pellets were collected after centrifugation of BALF and were suspended with RPMI 1640 (Sigma) in 96-well plates overnight. The non-adherent cells were washed away. The adherent cells were collected and washed three times with RPMI 1640 medium to acquire purified macrophages.

Since SC mainly induced M2 macrophages, we used the mouse macrophage cell line RAW 264.7 (Procell, China) in subsequent studies. The cells were incubated in Dulbecco’s modified Eagle’s medium (DMEM) (Gibco, Grand Island, USA) containing 10% fetal bovine serum (FBS, Sigma, cat#12107C) and 1% penicillin-streptomycin in a humidified incubator with 5% CO_2_ at 37°C. Macrophages were cultured with 5 ng/mL lipopolysaccharide (Sigma, cat#SMB00610) and 100 U/mL IFN-γ (Sigma, cat#SRP3058) for 24 h for M1 polarization. To obtain anti-inflammatory macrophages (M2), macrophages were cultured with 10 ng/mL IL-4 (Sigma, cat#SRP3211) for 24 h.

### Mouse primary airway epithelial cell culture

Mouse primary airway epithelial cells were incubated in Dulbecco’s modified Eagle’s medium (DMEM) (Gibco, Grand Island, USA) containing 10% FBS and 1% penicillin-streptomycin in a humidified incubator with 5% CO_2_ at 37°C.

### Isolation and identification of exosomes

Exosomes were extracted from macrophages, as reported previously [22]. Briefly, macrophages were cultured in the exosome-depleted medium for 72 h. Then, the cell medium was harvested and centrifuged at 300 g for 10 min at 4°C and the media were filtered using a 0.22 μm filter. Finally, the media was ultracentrifuged at 120000 g for 70 min at 4°C and exosomes were collected. The exosomes were resuspended in 50 μl of PBS for the following experiments. Subsequently, the isolated exosomes were determined by transmission electron microscopy (TEM), particle analyzer, and specific protein markers. Exosomes were fixed with 1% buffered glutaraldehyde for 10 min. The 20 μL of exosomes were added to formvar carbon-coated 300-mesh copper electron microscopy grids (Agar Scientific Ltd., Stansted, UK) and allowed to stand for 5 min. Then 2% uranyl oxalate was added to counterstain exosomes at room temperature. After the grids were washed three times with PBS, the exosomes were photographed using TEM (Hitachi H7500 TEM, Tokyo, Japan). The exosome particle size analysis was conducted by nanoparticle tracking analysis (NTA) with ZetaView PMX 110 (Particle Metrix, Meerbusch, Germany). The CD63 was applied to identify exosomes via western blot analysis.

### Immunofluorescence

Macrophages were seeded on cell slides and incubated overnight. The cells were fixed with 4% paraformaldehyde and permeabilized with 0.3% Triton X-100. Next, the cells were blocked with 1% bovine serum albumin (GIBCO, cat#23208) and were incubated with primary antibodies against CD68 (1:1000, Santa Cruz, cat#sc-52998) and Arg-1 (1:500; Cell Signaling Technology, 93668S) at 4°C overnight. After washing with PBS, the cells were incubated with second antibodies (1:1000, Abcam, cat#ab150117) and DAPI (Abcam, cat#ab188804) at room temperature for 1 h. Images were acquired with a fluorescence microscope (Carl Zeiss, Oberkochen, Germany).

### Flow cytometry analysis

The ratio of CD163^+^/CD68^+^ was detected using flow cytometry. After reaching 70–80% confluence, the cultured cells were trypsinized and incubated with monoclonal antibodies, including B525-FITC-H CD163^+^ (Abcam, cat#ab182422) and Y585-PE-H CD68^+^ (Santa Cruz, cat#sc-52998) for 30 min at 4°C in the dark. Flow cytometry analysis was conducted using a FACSCalibur^™^ flow cytometer (BD Biosciences, NJ, USA), and the data were analyzed using FlowJo software (TreeStar, Ashland, OR, USA).

### Cell transfection

The miR-30b-5p mimics and miR-30b-5p inhibitors were synthesized by YinBio Technology (Shanghai, China). The macrophages were transfected using Lipofectamine 2000 (Invitrogen, Carlsbad, CA, USA) in light of the manufacturer’s protocol.

### Transmission electron microscopy (TEM)

TEM was used to observe the effect of macrophage derived-exosomes on pyroptosis of airway epithelial cells. The airway epithelial cells cultured by macrophage exosomes were fixed with 2% glutaraldehyde at 4°C overnight. Afterward, the cells were treated with 1% osmium tetroxide, stained with 1% uranyl acetate, dehydrated with gradient ethanol, and embedded in epoxy resin. Ultrathin tissue sections (60 nm) were cut with an ultracut microtome (Leica; Solms, Germany) and stained with uranyl acetate and lead citrate. Finally, cell morphology and subcellular structures were observed.

### Airway epithelial cells internalize macrophage derived-exosomes and M2 macrophage derived-exosomal miR-30b-5p

According to the manufacturer’s protocol, macrophage derived-exosomes were labeled with the fluorochrome DiI (Beyotime, cat#C1036). Airway epithelial cells were incubated with DMEM containing DiI-labeled exosomes for 24 h. After washing the cells, DAPI was used to stain the nucleus, and the results were analyzed with fluorescence microscopy (Nikon).

M2 macrophage was transfected with Cy3-labeled (Beyotime, cat#A0516) miR-30b-5p mimics, and exosomes were isolated. Airway epithelial cells were incubated with M2Φ-Exos transfected with Cy3-labeled miR-30b-5p mimics for 24 h. Cells were observed by fluorescence microscopy (Nikon) to internalize miR-30b-5p-containing exosomes.

### The effects of exosomes on asthmatic mice

The asthma mouse model was generated in the same method as described above. We first injected DiI-labeled exosomes into the mice by the tail vein and collected the mouse lung tissues after 24 hours to verify that exosomes could locate at the lung tissues. Subsequently, a total of 20 mice with SA treatment were randomly assigned into four groups: SA group, SC group (SA mice were treated with SC), SC plus NC-Exos group (SA mice were treated with SC and NC-M2Φ-Exos), and SC plus miR-30b-5p-Exos group (SA mice were treated with SC and miR-30b-5p overexpressed M2Φ-Exos) (*n* = 5). Exosome therapy referred to the literature Φ-Exos treatment, and the mice were treated with 20 μg M2Φ-Exos by tail vein twice a week reported by Li et al. [11]. Mice received M2 for 3 weeks, starting one week before model construction. Lung tissues and BALF were collected at the end of the experiment and stored at −80°C until further experiments.

### Transcriptome sequencing

The total RNA of airway epithelial cells from NC and miR-30b-5p mimics was extracted and prepared for transcriptome sequencing. Then, the adapters were added to both ends of the primers and amplified the cDNA library (Colibri Library Amplification Master Mix, Thermo, USA). Subsequently, the constructed cDNA library was qualified by Agilent 2100 Bioanalyzer and ABI Step One Plus real-time PCR System and sequenced on Illumina HiSeq 2500 platform (Illumina, San Diego, CA, USA) with 150 bp paired-end run. FastQC qualified the raw reads (http://www.bioinformatics.babraham.ac.uk/projects/fastqc/) and retrieved filtering data was mapped to the mice reference genome (GRCm38). To obtain gene profiles that differently expressed between mimics NC and miR-30b-5p mimics, we set a standard of log2FC > 1 or < -1 and FDR < 0.05. Gene Ontology (GO) database and Kyoto encyclopedia of genes and genomes (KEGG) analysis were performed to discover the functional role of target genes.

### Haematoxylin and eosin (H&E) staining

The lung tissues of mice were collected and immediately fixed with 4% formaldehyde solution for 48 hours. Then the tissues were dehydrated conventionally and embedded in paraffin. Subsequently, the tissues were cut into 4 μm thick sections for H&E staining. Pathological changes were observed using an optical microscope (BX-43; Olympus, Tokyo, Japan).

### Enzyme-linked immunosorbent assay (ELISA)

According to the manufacturer’s protocol, the levels of IL-1β (Shanghai Enzyme-Linked Biotechnology, cat#ml301814), TNF-α (Cayman Chemical, cat#500850), and IL-10 (Shanghai EnzymeLinked Biotechnology, cat#ml037873) were detected by indicated ELISA assay kits (Cayman Chemical, Michigan, USA). The absorbance value of each well was detected at 450 nm using the Microplate Reader (Thermo Scientific, NY, USA).

### Western blotting analysis

Total protein was extracted using RIPA lysis buffer. In short, the cells were centrifuged at 14,000 g for 10 min at 4°C, and the supernatant was collected. The total protein concentration was detected by the BCA protein assay kit (Thermo Scientific, cat#23225). Samples were segregated by 6%–10% SDS-PAGE and transferred onto PVDF membranes. Subsequently, the membranes were blocked with 5% non-fat milk at room temperature for 1 h and were incubated with primary antibodies against CD45 (1:1000, Abcam, cat#ab208022), CD68 (1:1000, Santa Cruz, cat#sc-52998), iNOS (1:200, Santa Cruz, cat#sc-7271), CD163 (1:1000, Abcam, cat#ab182422), NLRP3 (1:1000, Abcam, cat#ab214185), caspase-1 (1:1000, Santa Cruz, cat#sc-56036) GAPDH (1:10000, Proteintech, cat#60004-1-Lg) at 4°C overnight. Membranes were washed and incubated with horseradish peroxidase-conjugated secondary antibody (1:2000; Proteintech, SA00001-1) for 1.5 h at room temperature. Finally, the data were analyzed by Image J software (v5.2.1).

### Immunohistochemistry

Lung tissues were fixed with 4% formaldehyde, embedded in paraffin, and made into 3-μm sections. Then the sections were deparaffinized and dehydrated in alcohol. Subsequently, antigen retrieval was conducted by boiling the sections in 0.01 M sodium citrate buffer. The sections were blocked with 5% BSA and incubated at 4°C overnight with primary antibodies against NLRP3 (1:1000, Abcam, cat#ab214185) and caspase-1 (1:1000, Santa Cruz, cat#sc-56036), followed by incubation with a secondary antibody (1:1000, Beyotime, cat#A0208) for 1 h at room temperature. In the end, we performed 3, 3’-diaminobenzidine tetrahydrochloride (DAB) and hematoxylin staining. Images were photographed with a microscope (Olympus, Tokyo, Japan).

### Gene expression analysis

Total RNA was extracted with TRIzol reagent (Invitrogen Life Technologies, Inc. cat#15596018) following the manufacturer’s protocol. The concentration and purity of RNA were measured by a microspectrophotometer (Tiangen Biotech Co., Ltd.). Total RNA was reverse transcribed into First-strand cDNA using RevertAid First Strand cDNA synthesis kit (ThermoScientific, cat# K16225). qRT-PCR was conducted using FastStart Universal SYBR Green Master mix (Roche, cat#04913850001) with QuantStudio 6 Flex Real-Time PCR System (Thermo Fisher Scientific, Inc). All primers used in this study were synthesized by Yingbai Biotech (Shanghai, China) and shown in Supplementary Table 1. U6 and GAPDH were served as internal reference genes, and the relative gene expression was calculated by the 2^−ΔΔCq^ method.

### Data analysis

The sample size was determined by experience and based on similar work in the literature. The investigators who performed mouse experiments were not blinded. Statistical analysis was carried out using SPSS 17.0, and the data were presented as the mean ± standard deviation (SD). Statistically significant differences were determined by the two-tailed Student’s *t*-test. (two groups) and one-way analysis of variance followed by Tukey’s post hoc test (more than two groups). The standard for statistical significance was set at *P* < 0.05.

### Ethics approval

All experimental procedures were approved by the Ethical Committee and the Animal Experimental Committee of Shanghai Municipal Hospital of Traditional Chinese Medicine, Shanghai University of Traditional Chinese Medicine.

## RESULTS

### SC induces M2Φ-Exos

**Figure 1:**
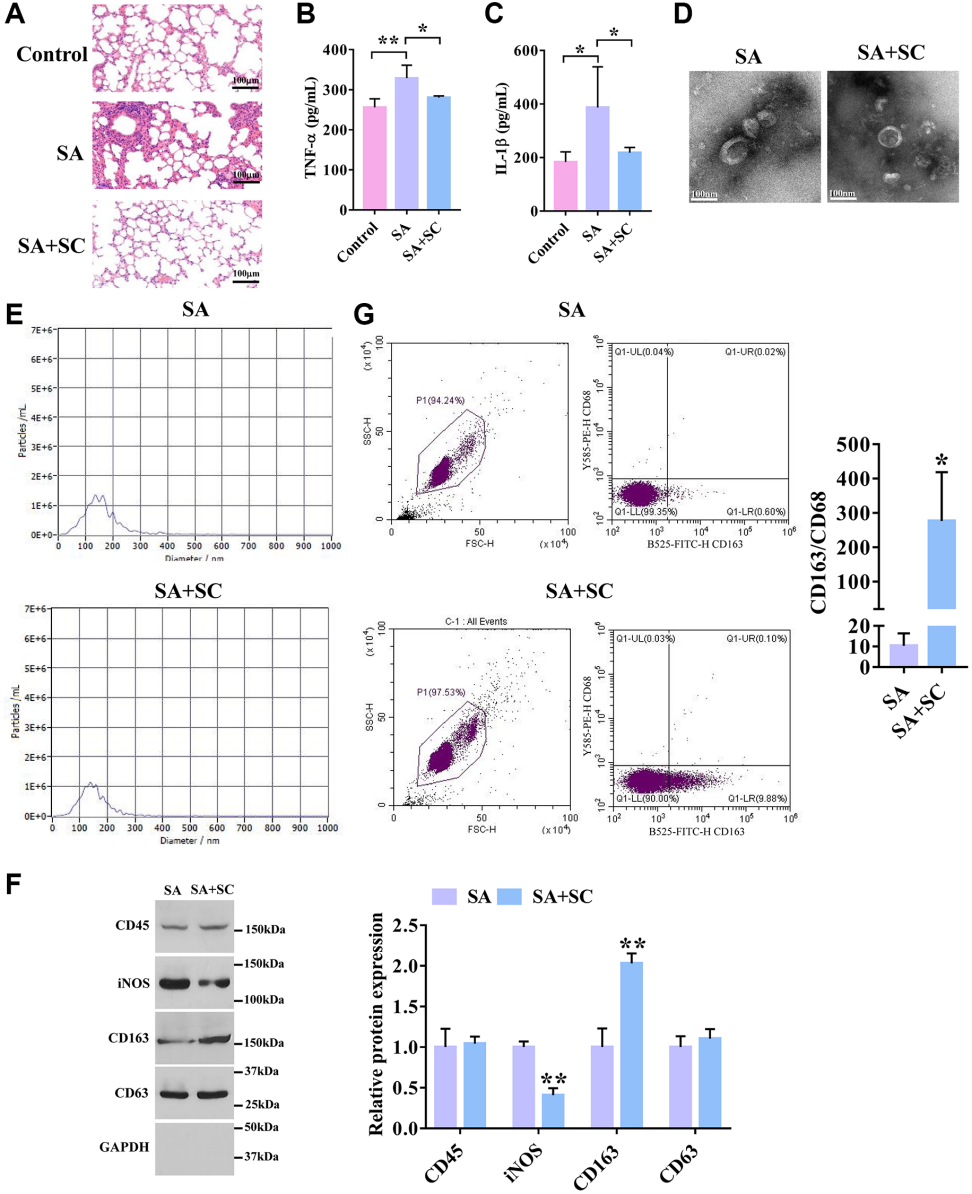
**SC improves severe asthma.** (**A**) H&E staining of lung tissues in the SA mouse model treated with SC. Scale bars = 100 μm. (**B**) The expression level of TNF-α in lung tissues was detected by ELISA assay. (*n* = 5) (**C**) The expression level of IL-1β in lung tissues was detected by ELISA assay. (*n* = 5) (**D**) The shape and size of the particles were evaluated using transmission electron microscopy. Representative pictures were shown. Scale bars = 100 nm. (**E**) Diameter distribution and particle concentration of exosomes were observed by nanoanalysis. (**F**) The expression levels of exosome markers and macrophage markers were detected by Western blotting. (**G**) Flow cytometry analysis of CD163+/CD68+ ratio. ^*^P < 0.05; ^**^P < 0.01.

To investigate the effects of SC on SA, the SA mouse model was generated and treated with SC. As shown in Figure 1A, the bronchial mucosa and alveoli epithelium was not deformed in the control group, and there was no inflammatory cell, whereas a large number of goblet cells were gathered in the alveoli in the SA group, and the alveolar walls were congested and edema. Also, a large number of inflammatory cells were observed. Furthermore, the epithelial cells of the bronchial mucosa were deformed. Conversely, after SC treatment, slight hyperemia, redness, and swelling of alveoli were seen, and a few inflammatory cells were observed in alveoli. Compared with the SA group, the number of inflammatory cells and the edema of the bronchial mucosa epithelium were significantly reduced. The ELISA assay revealed that SC significantly decreased the expression levels of TNF-α and IL-1 in the SA mouse model (Figure 1B and 1C).

Subsequently, to determine what kind of cell-derived exosomes were mainly regulated by SC, the exosomes from bronchoalveolar lavage fluid of the SA and SC groups were isolated. TEM images indicated that the morphology of exosomes exhibited a near-spherical shape (Figure 1D). The nanoparticle tracking analysis showed that the particle size was between 50 and 200 nm (Figure 1E). Moreover, SC had no significant effect on the morphology and size of exosomes, and the concentration of exosomes in BALF was not affected by SC. As shown in Figure 1F, the expression of exosome marker CD63 and the white blood cell marker CD45 had no significant difference between the two groups. The expression of the M1 macrophage marker, iNOS, was significantly decreased in the SC group compared with the SA group, whereas SC remarkably promoted the expression of the M2 macrophage marker CD163 compared with the SA group. Similarly, flow cytometry analysis showed that the CD163+/CD68+ ratio was much higher in exosomes of the SC group than the SA group (Figure 1G). Altogether, these results indicate that SC could induce M2Φ-Exos to alleviate SA.

### M2Φ-Exos inhibit airway epithelial cell pyroptosis

**Figure 2:**
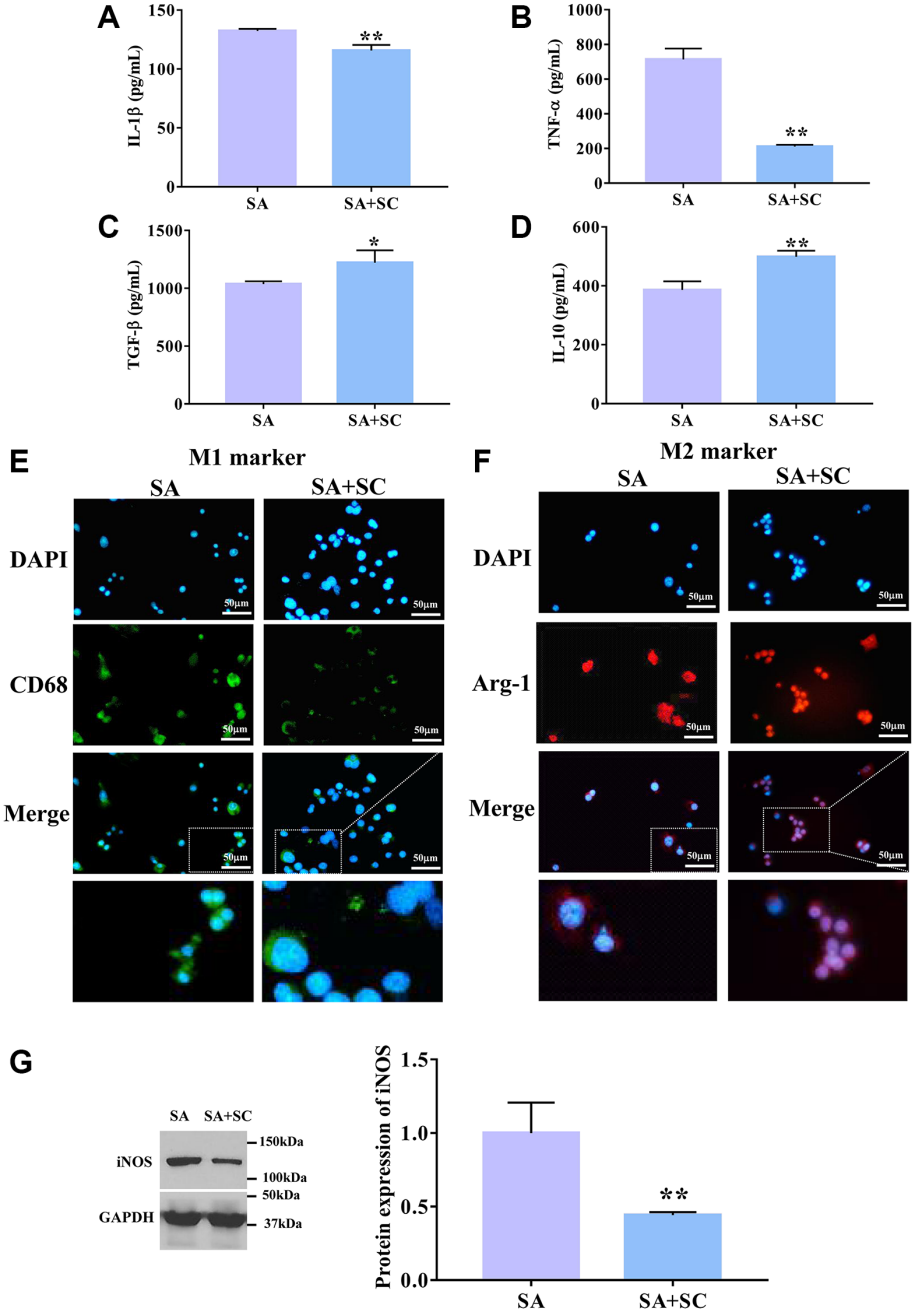
**SC induces M2 macrophage.** The expression level of (**A**) IL-1β, (**B**) TNF-α, (**C**) TGF-β, and (**D**) IL-10 was detected by ELISA assay. Immunofluorescence determined the expression level of M1 marker (**E**) and M2 marker (**F**). Scale bars = 50 μm. (**G**) The protein expression level of iNOS was determined by Western blotting. ^*^P < 0.05; ^**^P < 0.01.

To explore whether SC promoted the increase of M2Φ-Exos, we isolated and detected macrophages in SC-treated BALF. As shown in Figure 2A–2D, the IL-1β and TNF-α expression levels were significantly decreased, while IL-10 and TGF-β levels were dramatically increased in the SC group compared with the SA group. Further, the M2Φ marker, Arg-1, was enhanced, while the M1Φ marker CD68 was reduced in the SC group compared to the SA group (Figure 2E and 2F). We also found that the iNOS expression level was markedly reduced in the SA+SC group compared to the SA group (Figure 2G). These results suggest that SC promotes the polarization of macrophages towards M2Φ in SA mice, which mediates the increase of M2Φ-Exos.

**Figure 3:**
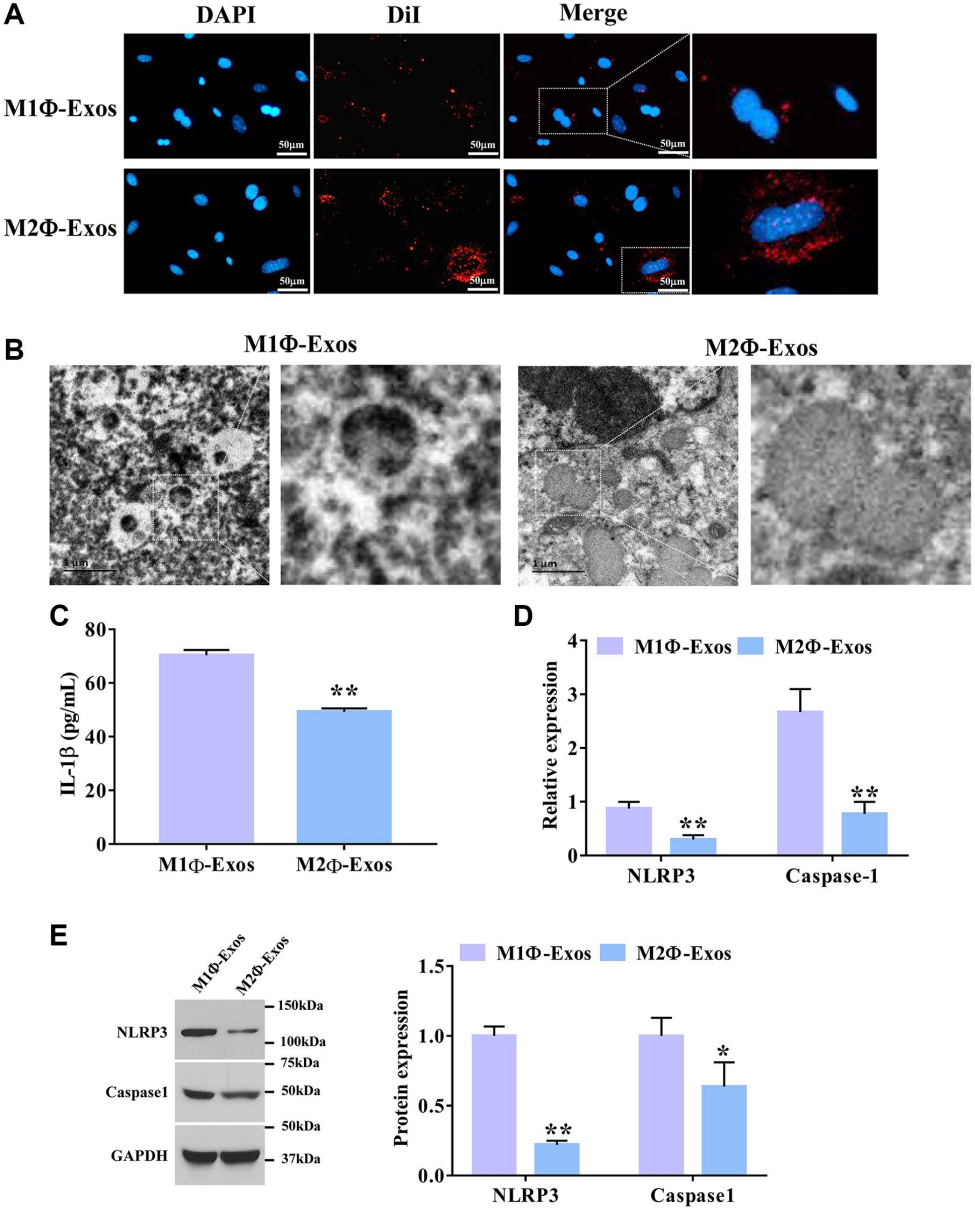
**M2Φ-Exos regulate airway epithelial cell pyroptosis.** (**A**) M2Φ-Exos internalization was observed using a laser scanning confocal microscope. Scale bars = 50 μm. (**B**) The changes of pyroptosis were observed by electron microscope. Scale bars = 1 μm. (**C**) The expression level of IL-1was detected by ELISA assay. (*n* = 3) (**D**) The relative expression levels of NLRP3 and caspase-1 were determined by qPCR. GAPDH was used as an internal control. (*n* = 3) (**E**) The protein expression levels of NLRP3 and caspase-1 were determined by Western blotting. ^*^P < 0.05; ^**^P < 0.01. Abbreviations: M1Φ-Exos: M1 macrophage-derived exosomes; M2Φ-Exos: M2 macrophage-derived exosomes.

Next, we further investigated the effect of SC-induced M2Φ-Exos on airway epithelial cells. To observe the process of exosome internalization by airway epithelial cells, the DiI-labeled M1Φ- and M2Φ-Exos were co-cultured with airway epithelial cells for 24 h. As shown n Figure 3A, more red fluorescence was observed in airway epithelial cells co-cultured with DiI-labeled M2Φ-Exos compared with the M1Φ-Exos. We also found that M2Φ-Exos caused less cell swelling and fewer bubbles blowing from the plasma membrane than the M1Φ-Exos (Figure 3B). ELISA assay showed that M2Φ-Exos markedly decreased the expression level of IL-1β in airway epithelial cells (Figure 3C). Additionally, the M2Φ-Exos significantly decreased the mRNA expression levels of NLRP3 and caspase-1 compared with the M1Φ-Exos (Figure 3D). Finally, we found that M2Φ-Exos dramatically decreased the protein expression levels of NLRP3 and caspase-1 compared with the M1Φ-Exos (Figure 3E). Taken together, these findings suggest that M2Φ-Exos inhibits airway epithelial cell pyroptosis.

### M2Φ-Exos carry miR-30b-5p to suppress airway epithelial cell pyroptosis

**Figure 4:**
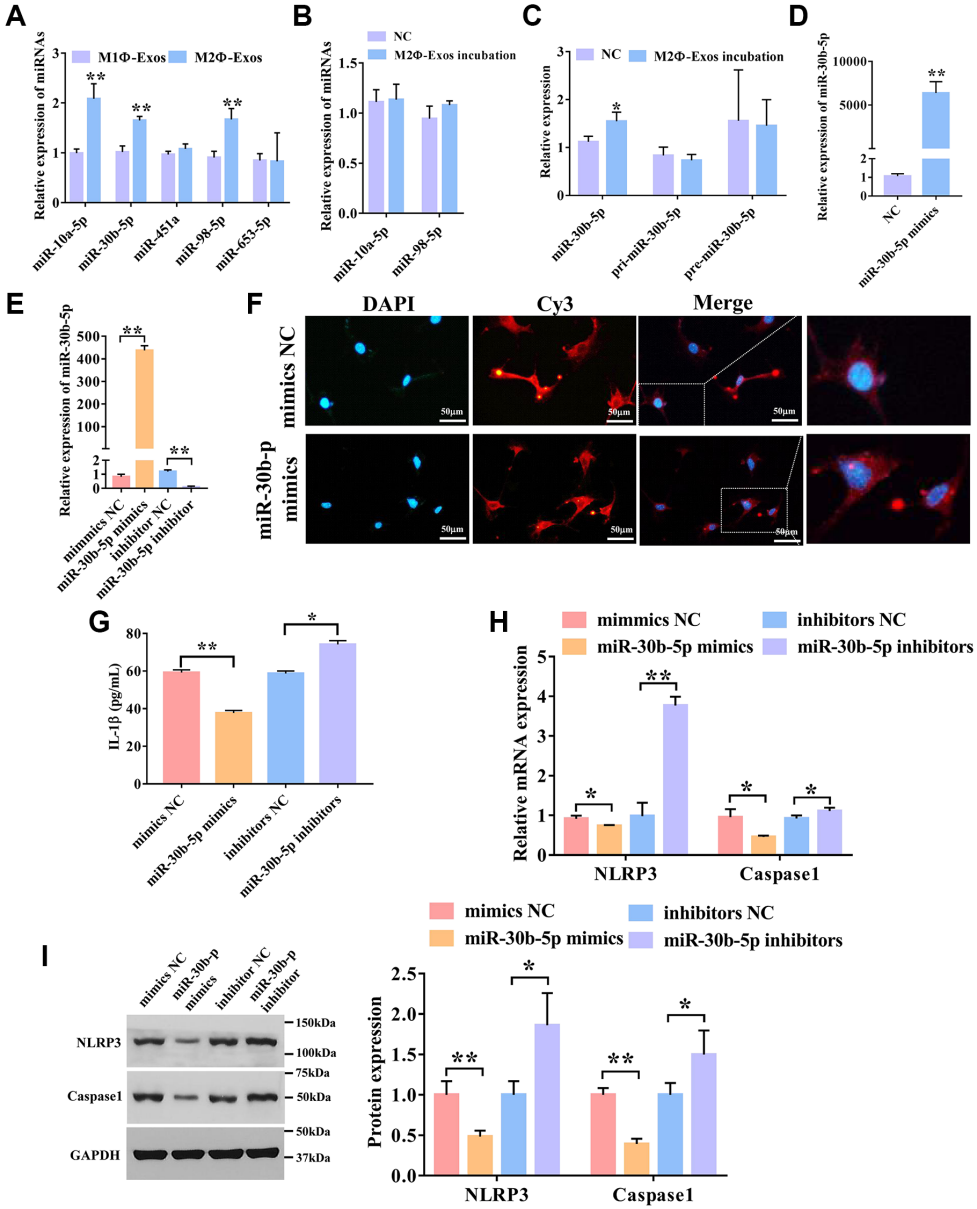
**M2Φ-Exos transfer miR-30b-5p to regulate airway epithelial cell pyroptosis.** (**A**) The expression levels of miRNAs in M2Φ-Exos. qRT-PCR was used to detect five candidate miRNAs. U6 was used as an internal control. (*n* = 3) (**B**) The expression levels of miRNAs in airway epithelial cells incubated with M2Φ-Exos. (*n* = 3) (**C**) The expression level of miR-30b-5p in airway epithelial cells incubated by M2Φ-Exos. (*n* = 3) (**D**) The expression level of miR-30b-5p in M2Φ-Exos transfected with miR-30b-5p mimics. (*n* = 3) (**E**) Overexpression and interference efficiency of miR-30b-5p were detected by qRT-PCR. (*n* = 3) (**F**) M2Φ-Exos containing miR-30b-5p internalization was observed with a fluorescence microscope. Scale bars = 50 μm. (**G**) The expression level of IL-1β was measured s by ELISA assay in airway epithelial cell. (*n* = 3) (**H**) The expression levels of NLRP3 and caspase-1 were determined by qPCR. (*n* = 3) (**I**) The protein expression level of NLRP3 and caspase-1 was detected by Western blotting. ^*^P < 0.05; ^**^P < 0.01.

Previously, we screened differentially expressed miRNAs in exosomes from BALF of SC treated and untreated asthma mice using RNA sequencing [6]. We screened miRNAs that were highly expressed in BALF-derived exosomes after SC therapy. We found that these miRNA target genes are related to pyroptosis (Supplementary Figure 1). We selected the five most differentially expressed miRNAs and confirmed their expressions in M1Φ-and M2Φ-Exos using qPCR. As shown in Figure 4A, the expression levels of miR-10a-5p, miR-30b-5p, and miR-98-5p were markedly up-regulated in the M2Φ-Exos relative to the M1Φ-Exos (Figure 4A). We also incubated airway epithelial cells with M2Φ-Exos and examined these miRNAs, and found that only miR-30b-5p was dramatically up-regulated in airway epithelial cells (Figure 4B and 4C). Therefore, we further investigated the effect of miR-30b-5p. To explore whether miR-30b-5p was carried by M2Φ-Exos, we detected the expression of pir-miR-30b-5p and pre-miR-30b-5p after incubation with exosomes. We found that there was no significant difference in the expression of pri-miR-30b-5p and pre- miR-30b-5p (Figure 4C). The expression of miR-30b-5p was remarkably up-regulated in M2Φ-Exos transfected miR-30b-5p mimics (Figure 4D). Additionally, overexpression and interference efficiency of miR-30b-5p was evaluated by qRT-PCR, and results revealed that miR-30b-5p was significantly up-regulated in M2Φ-Exos transfected with miR-30b-5p mimics and down-regulated in M2Φ-Exos transfected with miR-30b-5p inhibitors (Figure 4E). Subsequently, to further confirm miR-30b-5p could be delivered to airway epithelial cells by M2Φ-Exos, airway epithelial cells were incubated with M2Φ-Exos transfected with Cy3-labeled miR-30b-5p mimics. The red fluorescence was clearly observed in airway epithelial cells (Figure 4F). Then, we treated the airway epithelial cells with M2Φ-Exos transfected miR-30b-5p mimics/inhibitors/NC to detect pyroptosis. As shown in Figure 4G, we found that M2Φ-Exos with overexpression of miR-30b-5p remarkably decreased the expression of IL-1β, while M2Φ-Exos with knockdown miR-30b-5p increased the expression of IL-1β compared to the M2Φ-Exos-NC. The qPCR showed that miR-30b-5p mimics markedly inhibited the expression of NLRP3 and caspase-1, while M2Φ-Exos with deficiency of miR-30b-5p enhanced the expression of NLRP3 and caspase-1 in airway epithelial cells (Figure 4H). In line with qPCR results, miR-30b-5p mimics dramatically reduced the protein expression of NLRP3 and caspase-1, whereas miR-30b-5p inhibitors markedly increased their protein expression (Figure 4I). Altogether, M2 macrophage-derived exosomal miR-30b-5p represses airway epithelial cell pyroptosis.

### MiR-30b-5p regulates target genes and signaling pathways in M2Φ-Exos

**Figure 5:**
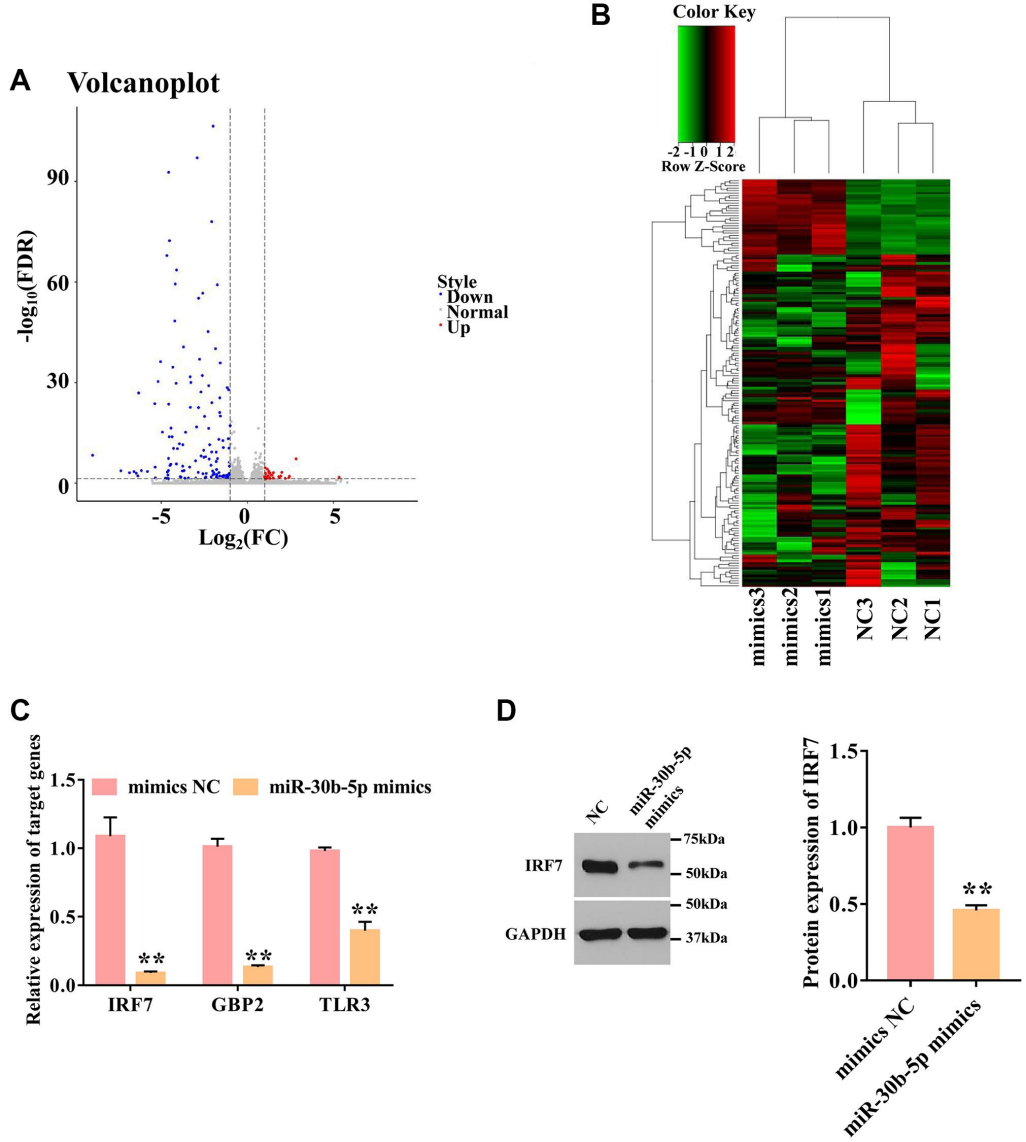
**Overexpression of miR-30b-5p down-regulates IRF7.** (**A**) Volcano plot of differentially expressed genes. The red dot represents up-regulated genes, and the blue dot represents down-regulated genes. (**B**) Cluster heatmap of differentially expressed genes. Each column represents a sample; the row shaded in red represents up-regulated genes, and shaded in green represents down-regulated genes. (**C**) Validation of 3 selected targeted genes by qPCR. GAPDH was used as an internal control. (*n* = 3) (**D**) The protein expression level of IRF7 was detected by Western blotting. ^**^P < 0.01.

To elucidate the key molecules and potential signaling pathways mediated by miR-30b-5p, RNA sequencing was performed in the airway epithelial cells transfected by miR-30b-5p mimics or NC. In total, 163 differentially expressed genes (DEGs) were identified, including 30 up-regulated DEGs and 133 down-regulated DEGs in the mimics group compared with the NC group (Figure 5A). The six samples were clustered closely to two groups, namely the mimics group, and the NC group, indicating a difference in target gene expression between the mimics group and the NC group (Figure 5B). To characterize the function of DEGs, GO and KEGG pathway analyses were performed. For GO analysis, the target genes were mainly participated in the immune system process, innate immune response, immune response, and metabolic process (Supplementary Figure 2A). KEGG enrichment analysis revealed that target genes were mainly involved in the RIG-I-like receptor signaling pathway, chemokine signaling pathway, Jak-STAT signaling pathway, and NF-kappa B signaling pathway (Supplementary Figure 2B). Combined with the prediction results of miR-30b-5p target genes (Supplementary Figure 1), the down-regulated genes (IRF7, GBP2, and TLR3) were identified as their candidate genes. The qPCR demonstrated that miR-30b-5p mimics obviously inhibited the expression levels of IRF7, GBP2, and TLR3 compared with the mimics NC group (Figure 5C). Among them, IRF7 showed a most significant decrease. Thus, the role of IRF7 in SA was further explored. As shown in Figure 5D, miR-30b-5p mimics significantly blocked the protein expression of IRF7.

### M2Φ-Exos induced by SC carry miR-30b-5p to improve severe asthma

**Figure 6:**
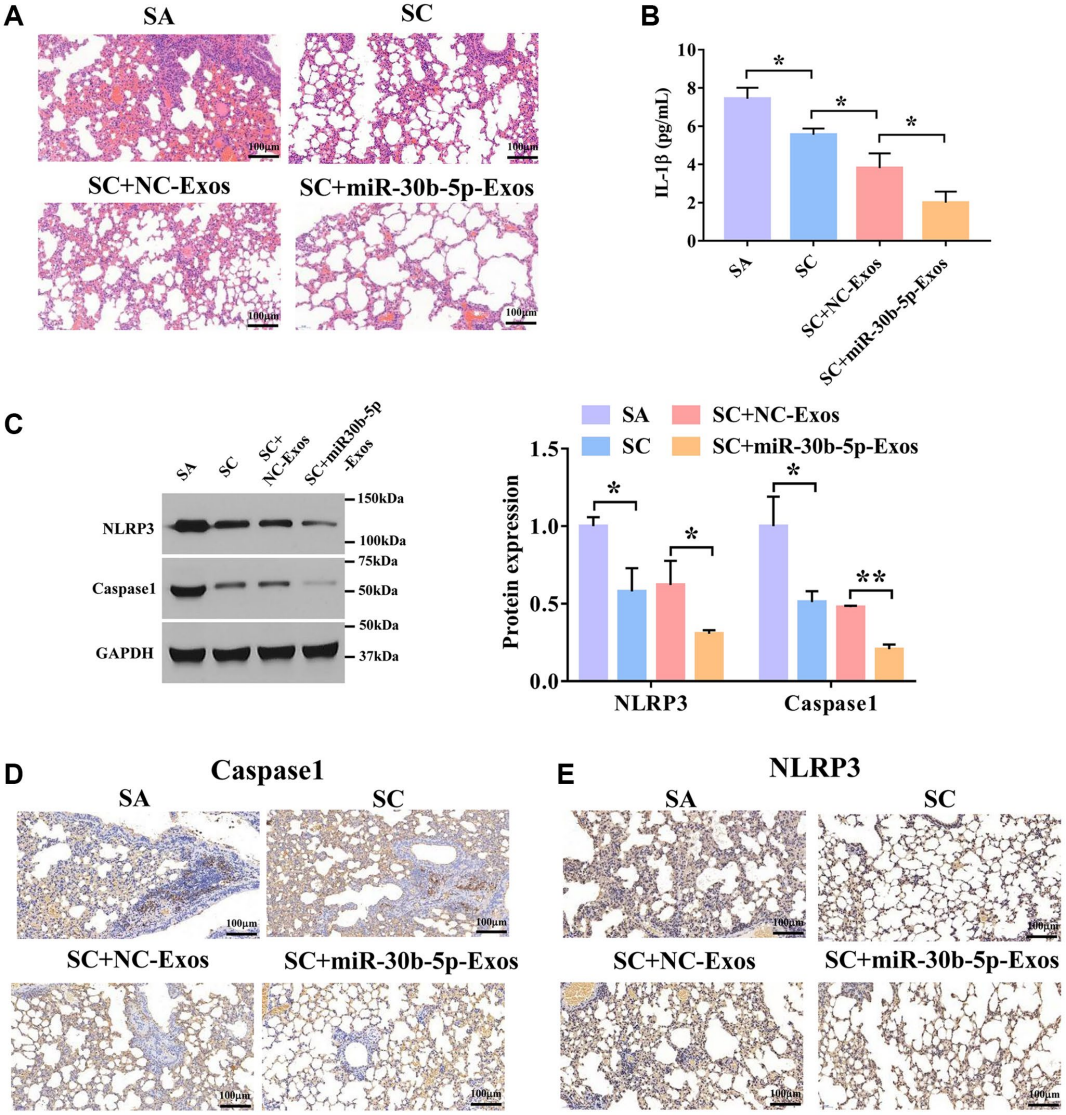
**M2Φ-Exos induced by SC carry miR-30b-5p to treat severe asthma.** (**A**) H&E staining of lung tissues in SA model treated with SC and Exos-miR-30b-5p. Scale bars = 100 μm. (**B**) The expression level of IL-1β in lung tissues was detected by ELISA assay. (*n* = 3) (**C**) The expression of NLRP3 and caspase-1 was detected by Western blotting. (**D**) and (**E**) The expression levels of caspase-1 and NLRP3 were measured by immunohistochemistry. Scale bars = 100 μm. ^*^P < 0.05; ^**^P < 0.01.

To investigate the effects of exosomal miR-30b-5p induced by SC in SA, we used the SA mouse model to validate the roles of exosomal miR-30b-5p in SA. To verify whether exosomes could reach the lung tissues, Dil-labeled exosomes were first injected into mice via tail vein, and the lung tissues of the mice were collected after 24 h. We found that both NC-Exos and miR-30b-5p-Exos injected mice had fluorescence, confirming that M2Φ-Exos could reach the lungs of mice (Supplementary Figure 3). As shown in Figure 6A, the tracheal mucosa was exfoliated and surrounded by a large number of inflammatory cells in the SA group. In the SC+NC-Exos and SC+ miR-30b-5p-Exos groups, the tracheal mucosa was intact without rupture, and the infiltration of surrounding inflammatory cells was less than that of the SC group. Compared with the SC+NC-Exos group, the number of inflammatory cells was reduced in the SC+miR-30b-5p-Exos group. In addition, the ELISA assay showed that SC markedly decreased the expression of IL-1β in lung lavage fluid compared with the SA group (Figure 6B). Meanwhile, SC+miR-30b-5p-Exos remarkably reduced the expression of IL-1β in lung lavage fluid compared with the SC+NC-Exos group. Furthermore, NLRP3 and caspase-1 protein expression was remarkably reduced in SC treated SA mice, and miR-30b-5p overexpressed M2Φ-Exos further enhanced the effect of SC (Figure 6C). Consistent with these results, SC significantly decreased NLRP3 and caspase-1 expression, and miR-30b-5p overexpressed M2Φ-Exos further blocked the expression of NLRP3 and caspase-1 with SC treatment (Figure 6D and 6E). Collectively, these results uncover that M2Φ-Exos carried miR-30b-5p attenuate SA via inhibiting airway epithelial cell pyroptosis.

## DISCUSSION

Previously, we discovered that SC induced the alternation of exosomal miRNAs in asthma through small RNA sequencing [6]. However, the role of SC-induced exosomes in SA progression and the related molecular mechanisms have not been explored. In the present study, we found that SC mainly induced changes in M2Φ-Exos by promoting the polarization of M2 macrophages, and then M2Φ-Exos carried miR-30b-5p to alleviate SA by inhibiting airway epithelial cell pyroptosis. Pyroptosis is a newly discovered type of programmed cell death related to inflammation [23]. Once pyroptosis is activated, inflammasome-mediated caspase-1 activation causes membrane rupture, followed by the release of pro-inflammatory cytokines, including IL-1β. Emerging evidence suggested that pyroptosis plays a critical role in developing multiple allergic diseases, especially SA [24]. Recent studies have confirmed that activation of NLRP3 inflammasome was significantly associated with the development of severe/refractory asthma using human and mouse asthma models [25]. Moreover, NLRP3 inflammasome-specific inhibitors can significantly prevent respiratory inflammation and airway hyperresponsiveness in a mouse model of severe allergic asthma [26]. In our study, we showed that SC induced the increase of M2Φ-Exos, and then M2Φ-Exos promoted airway epithelial cell pyroptosis. The increased expression of IL-1β often accompanies the process of cell pyroptosis. It has been reported that the serum IL-1β content was significantly increased in asthmatic patients [27]. Inhibition of IL-1β expression during asthma pathogenesis is promising therapeutic potential for treating asthmatic patients. Similarly, we also found that M2 macrophage exosomes significantly decreased the expression of IL-1 in airway epithelial cells. These results indicated that SC-induced M2Φ-Exos improve SA progression via regulating airway epithelial cell pyroptosis.

Exosomes are novel diagnostic markers of human diseases and ideal drug carriers and play an essential role in chronic airway inflammatory diseases, such as asthma and chronic obstructive pulmonary disease [28]. Deng et al. showed that M2 macrophage-derived exosomal miR-590-3p suppressed inflammatory signals and reduced mucosal damage induced by dextran sulfate sodium salt [29]. Also, Wu et al. uncovered that M2Φ-Exos carried lncRNA PVT1 to alleviate inflammation and protect experimental autoimmune encephalomyelitis mice [30]. In the current study, we found that the fibrosis in mouse lung tissues and the secretion of IL-1β in BALF were reduced by M2Φ-Exos. In accordance with our results, Dai et al. revealed that M2Φ-Exos alleviated myocardial ischemia/reperfusion injury via the inhibition of pyroptosis neonatal rat cardiomyocytes [31]. Collectively, these results demonstrated that M2Φ-Exos plays a protective role in SA by regulating airway epithelial cell pyroptosis.

A growing number of evidence suggests that miRNAs are closely associated with SA, and exosomes can deliver those miRNAs [32]. Previous studies reported that M2Φ-Exos carried miR-370 to inhibit inflammation and alleviate asthma progression by regulating the FGF1/MAPK/STAT1 axis [11]. miR-30b-5p was involved in the inflammatory response in multiple diseases, including acute lung injury and nonalcoholic fatty liver diseases [33, 34]. Here, we showed that miR-30b-5p was up-regulated in BALF of patients in the SC group, suggesting that miR-30b-5p influenced the pathogenesis of SA. In agreement with our findings, a previous study found that miR-30b-5p was participated in cannabinoid receptor 1-mediated activation of NLRP3 inflammasome in macrophages, and miR-30b-5p agomir decreased the expression of NLRP3, thereby attenuating liver inflammation [35]. Therefore, M2Φ-Exos could carry miR-30b-5p to inhibit inflammation and improve SA by regulating pyroptosis.

IRF7 was reported to play a pivotal role in regulating inflammatory response, cell differentiation, and apoptosis [36]. IRF7 has been shown to be involved in various diseases, including hepatitis C infection, systemic sclerosis, and pulmonary hypertension [37–39]. Deficiency of IRF7 impaired the expansion and function of type 2 innate lymphoid cells in the lung, inhibiting allergic airway inflammation [40]. In addition, IRF7 over-expression could remarkably promote pyroptosis in adipocytes, characterized by increasing caspase1 and IL-1β expression levels [41]. In our study, we identified the potential targets of miR-30b-5p and discovered that IRF7 was a candidate target of miR-30b-5p. Taken together, exosomal miR-30b-5p inhibits the pyroptosis during SA progression by regulating IRF7.

In summary, our study demonstrates that M2Φ-Exos induced by SC carry miR-30b-5p to suppress airway epithelial cell pyroptosis, thus alleviating SA progression by down-regulating IRF7 expression. These findings provide a deep insight into the regulatory mechanism of M2Φ-Exos induced by SC in SA progression, which may be a novel target for the treatment of SA.

## Supplementary Materials

Supplementary Figures

Supplementary Tables
