# Supplementary material for: Scorpion and centipede alleviates severe asthma through M2 macrophage-derived exosomal miR-30b-5p
Source: Aging (Albany NY). 2022 May 2;14(9):3921–40. doi: 10.18632/aging.204053 (PMC9134957; doi:10.18632/aging.204053)
Supplement: Supplementary Figures [file aging-14-204053-s001.pdf]

SUPPLEMENTARY FIGURES

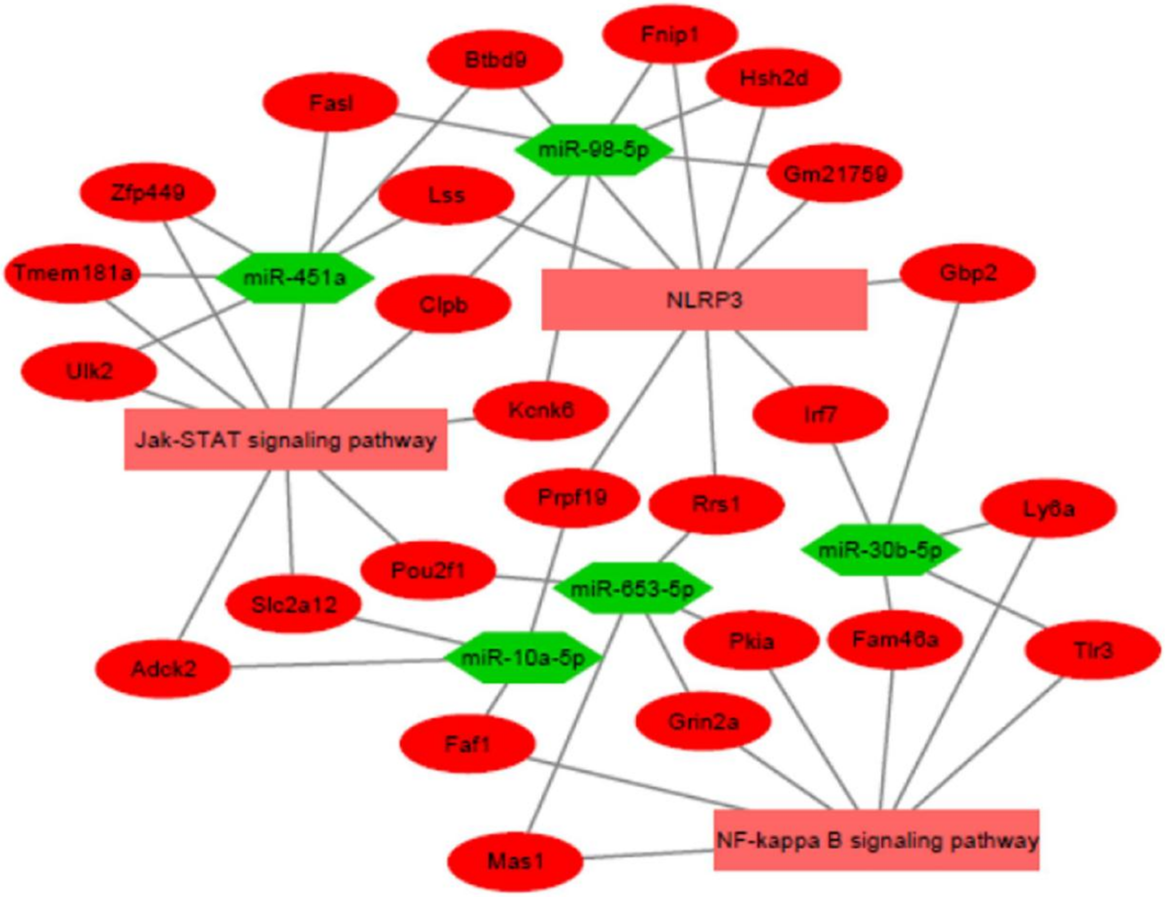

Supplementary Figure 1. The regulatory network of miRNAs and target genes and pathways. The hexagonal nodes represent miRNAs, the circular nodes represent mRNAs, and the quadrangular nodes represent pathways.

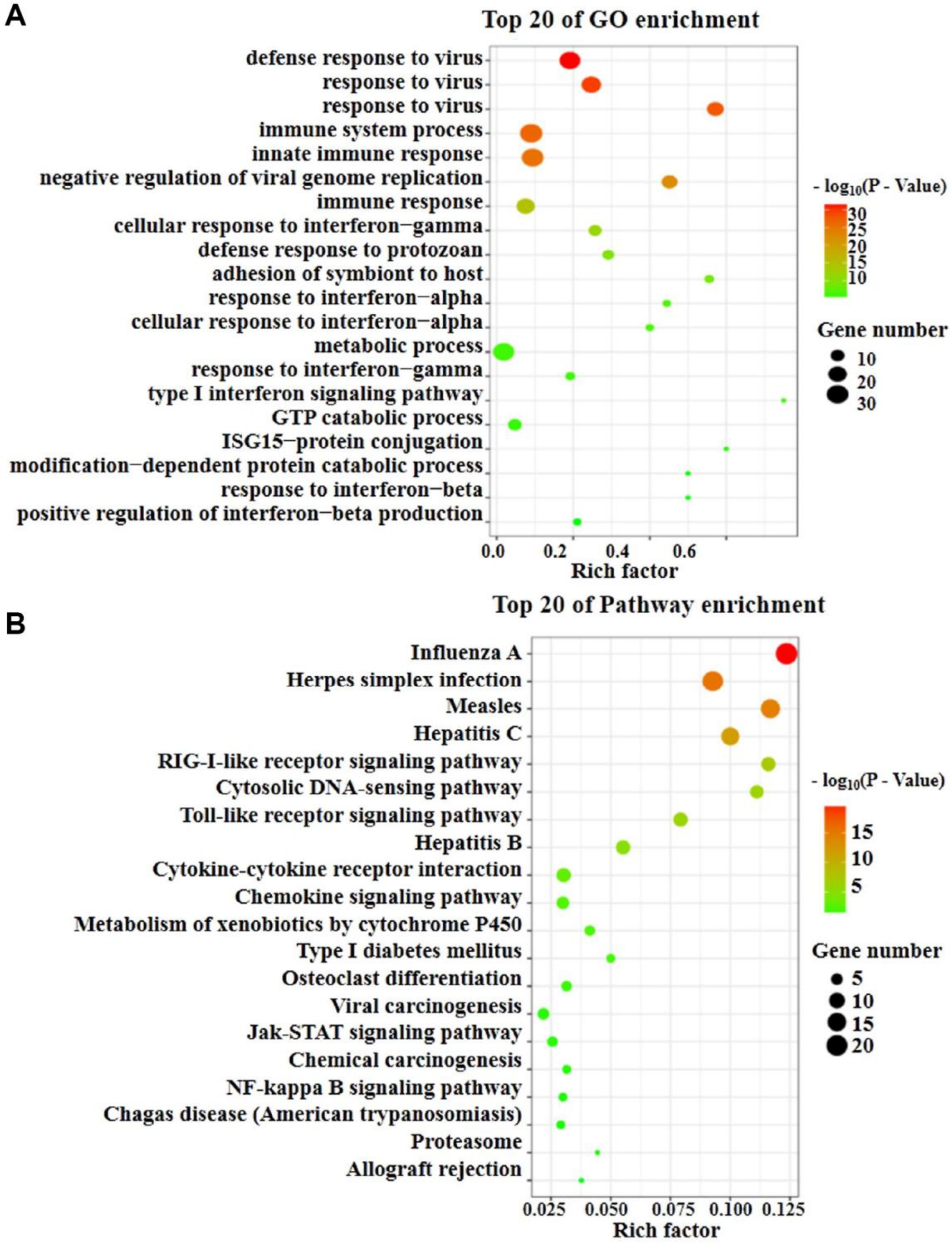

Supplementary Figure 2. GO and KEGG pathways enrichment based on differentially expressed genes. (**A**) The top 20 GO enrichment terms. The left represents GO terms, the right represents enrichment, and the size of the solid circle indicates the number of genes. (**B**) The top 20 KEGG enrichment pathways. The left represents the KEGG pathway, the right represents enrichment, and the size of solid circle indicates the number of genes.

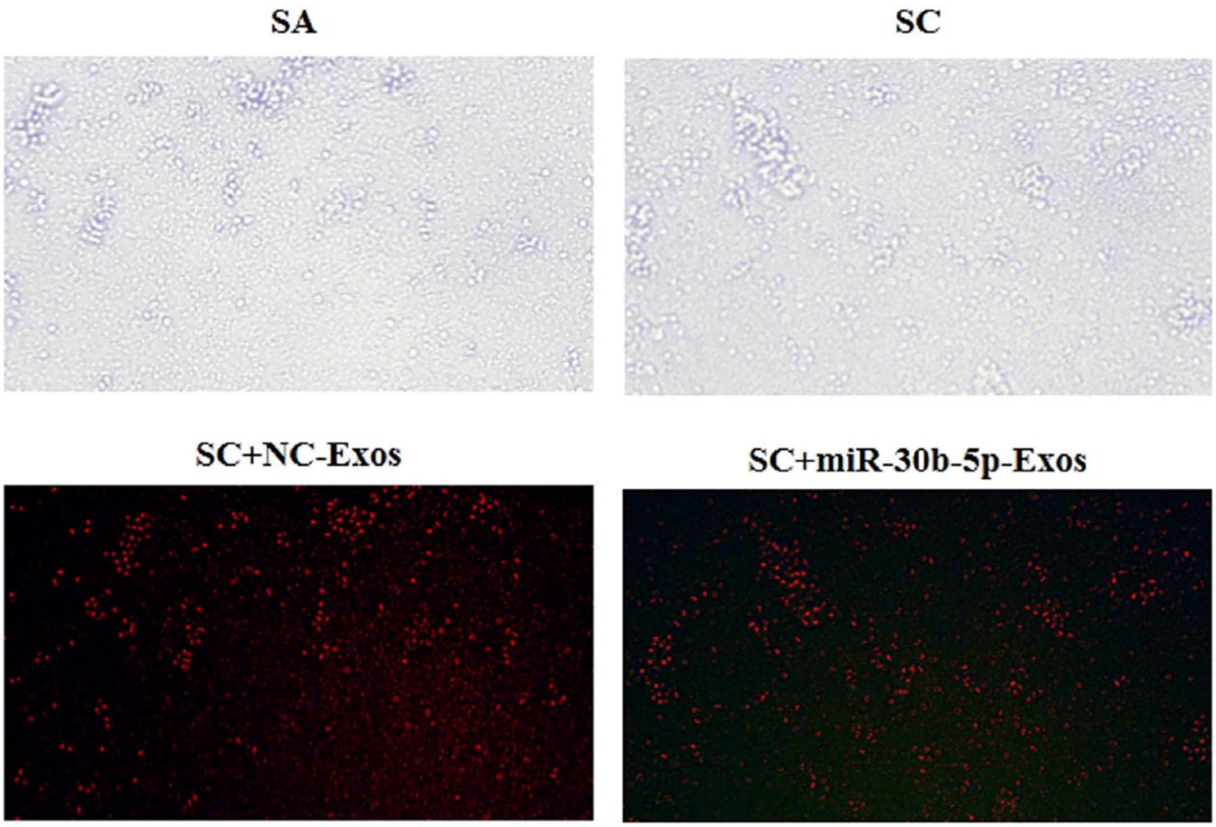

Supplementary Figure 3. Distribution of exosomes in lung tissues of mice. All images were captured at ×40 magnification.
