# Supplementary material for: Scorpion and centipede alleviates severe asthma through M2 macrophage-derived exosomal miR-30b-5p
Source: Aging (Albany NY). 2022 May 2;14(9):3921–40. doi: 10.18632/aging.204053 (PMC9134957; doi:10.18632/aging.204053)
Supplement: Supplementary Tables [file aging-14-204053-s002.pdf]

SUPPLEMENTARY TABLE

Supplementary Table 1. The primers and sequence used in this study.

Primers	Sequence (5′–3′)
U6-F	CGATACAGAGAAGATTAGCATGGC
U6-R	AACGCTTCACGAATTTGCGT
GAPDH-F	CAAAATGGTGAAGGTCGGTGT
GAPDH-R	GAGGTCAATGAAGGGGTCGTT
mmu-miR-653-5p-F	GCAGGTGTTGAAACAATCTCTA
mmu-miR-653-5p-R	AGTGCGTGTCGTGGAGTCG
mmu-miR-30b-5p-F	GCAGTGTAAACATCCTACACTCA
mmu-miR-30b-5p-R	AGTGCGTGTCGTGGAGTCG
mmu-miR-451a-F	CAGAAACCGTTACCATTACTGA
mmu-miR-451a-R	AGTGCGTGTCGTGGAGTCG
mmu-miR-98-5p-F	GGCGGGTGAGGTAGTAAGTTGT
mmu-miR-98-5p-R	AGTGCGTGTCGTGGAGTCG
mmu-miR-10a-5p-F	CGCAGTACCCTGTAGATCCGA
mmu-miR-10a-5p-R	AGTGCGTGTCGTGGAGTCG
mmu-pri-miR-30b-5p-F	CATACATGCGTTGGCTGGGA
mmu-pri-miR-30b-5p-R	CCTTTAGCTGGGCATGGTGAT
mmu-pre-miR-30b-5p-F	GTTTCAGTTCATGTAAACATCCTAC
mmu-pre-miR-30b-5p-R	AGTGCGTGTCGTGGAGTCG
NLRP3-F	TGGACCTCAGTGACAATGCC
NLRP3-R	GAGCTCAGAACCAATGCGAG
caspase-1-F	AAACGCCATGGCTGACAAGA
caspase-1-R	CGTGCCTTGTCCATAGCAGT
IRF7-F	CCCCAGCCGGTGATCTTTC
IRF7-R	CACAGTGACGGTCCTCGAAG
TLR3-F	AAAATCCTTGCGTTGCGAAGT
TLR3-R	TGTTCAAGAGGAGGGCGAATAA
GBP2-F	TGGGGTAGACGATTCCGCTAA
GBP2-R	AGAAGTGACGGGTTTTCCGTT
